# Perceptions of Chinese Patients Treated for Gynaecological Cancer about Sexual Health and Sexual Information Provided by Healthcare Professionals: A Qualitative Study

**DOI:** 10.3390/cancers13071654

**Published:** 2021-04-01

**Authors:** Ka Ming Chow, Carmen W. H. Chan, Bernard M. H. Law

**Affiliations:** The Nethersole School of Nursing, Faculty of Medicine, The Chinese University of Hong Kong, Hong Kong, China; kmchow@cuhk.edu.hk (K.M.C.); bernardlaw@cuhk.edu.hk (B.M.H.L.)

**Keywords:** Chinese, gynaecological cancer, qualitative study, sexual

## Abstract

**Simple Summary:**

Cancer patients undergoing treatment often experience undesirable symptoms including sexual problems, and are thus in need of high-quality sexual care and information from healthcare professionals on how to deal with sexual issues. In this study, we explored the types of sexual problems experienced by Chinese patients treated for gynaecological cancer, and their perception on how these problems can be addressed, so that strategies can be recommended to healthcare providers on how to augment the current sexual healthcare programmes for these patients. We demonstrated that Chinese patients were dissatisfied with their sexual lives owing to the loss of sexual function, sex drive, and femininity. They wished to receive information from healthcare professionals in which these professionals proactively initiate discussions on addressing sexual problems. Our findings suggested a need to implement programmes involving healthcare professionals from multiple disciplines, to enhance both the sexual and psychological wellbeing of these patients.

**Abstract:**

Patients treated for gynaecological cancer (GC) generally experience impaired sexual function. Research on their sexual life experiences and perceptions on the sexuality care they receive is warranted. This study aimed to examine the perceptions of Chinese patients treated for GC regarding the effects of cancer treatment on their sexual function and femininity, their relationships with their partners, and the adequacy of the sexual information received from healthcare professionals during treatment. Individual, semi-structured interviews were conducted with 21 Chinese patients treated for GC, collecting data on their perceptions regarding the effects of cancer treatment on their sexual lives, femininity, and relationships with partners; and their views about the quality of sexuality care received. Data were analysed using content analysis. Participants experienced impaired sexual function, reduced sex drive, and expressed dissatisfaction with their sex lives. They perceived a loss of femininity and poor body image. They desired more information about how to address sexual problems and opted to receive this information from female healthcare professionals in individual counselling sessions during which the professionals could initiate such discussions. Overall, Chinese patients treated for GC have concerns about multiple sexual issues and a strong desire for information about strategies to address these issues. Nurse-led interventions should be implemented via a shared care approach to enhance patients’ awareness about managing their sexual and psychological symptoms.

## 1. Introduction

Patients with gynaecological cancer (GC), which encompasses cervical, uterine, and ovarian cancers, often undergo curative treatments such as hysterectomy, chemotherapy, and radiotherapy during their rehabilitation. Even after completing treatment, patients continue to suffer from associated side effects that negatively affect their quality of life (QOL) and interfere with their daily lives [1]. Sexual dysfunction is one of the most common side effects experienced by patients treated for GC [2].

Previous studies have suggested that patients treated for GC generally have a higher risk of developing sexual dysfunction as a long-term effect of cancer treatment [3]. For example, patients treated for cervical cancer exhibit various aspects of impaired sexual function, including vaginal narrowing or shortening and reductions in sexual activity and sexual desire [4,5]. In particular, a reduction in sexual desire has been noted as one of the most common sexual problems among patients who completed GC treatment [6]. These individuals often experience difficulties related to sexual arousal and sexual satisfaction, and this loss of sex drive is thought to have detrimental effects on their wellbeing [7]. These patients have also reported decreases in vaginal elasticity and lubrication after the completion of treatment, and these effects have been reported to cause pain during sexual intercourse [8]. As a result, these sexual problems may contribute to the avoidance of sexual behaviour by these patients [9]. Moreover, they frequently report a worsening of body image [10]. These concerns likely have a negative effect on these patients’ sexual desire, function, and satisfaction [11], and may also lead to psychological issues, including distress, anxiety, and depression [12]. Notably, these signs of sexual dysfunction have been shown to reduce the patients’ QOL [13,14].

Given the negative effects of cancer treatment on sexual health, patients treated for GC often wish to receive adequate sexual information from healthcare providers and seek advice about strategies to promote and maintain sexual health during rehabilitation. Indeed, one study indicated that such patients primarily desire information services that can help them obtain sexual information from healthcare professionals through face-to-face discussions or written materials [15]. In another study, a significant proportion of patients treated for cervical cancer (51%) reported a desire for information on sexual issues [16]. These data demonstrate that the provision of information to address sexual concerns should constitute an integral component of the care provided to patients treated for GC.

Sexual health is identified as an integral component of any cancer survivorship programme [17]. Several models have been proposed to guide the development of programmes that seek to improve cancer patients’ QOL. The shared care model, as described by Oeffinger and McCabe [18], is commonly adopted to provide care for patients with chronic diseases. The model stipulates that healthcare professionals from different disciplines should be involved in providing care for patients that enables them to address their multiple health issues. Indeed, shared care provision has been demonstrated as an effective approach to enhance patient satisfaction among cancer patients [19]. It therefore has the potential to be implemented as part of the existing community cancer survivorship care programmes. However, further information regarding patients’ symptoms, especially those related to sexual issues, which are sensitive and private, is needed to determine the types of healthcare personnel who should be involved in the delivery of such care.

Although considerable information is available regarding the sexual issues of patients treated for GC, these data have been derived largely from studies conducted in Western countries. In contrast, few studies have been conducted among Chinese patients treated for GC. Notably, cultural differences among Eastern and Western societies indicate that these Chinese patients are likely to have different health needs that should be addressed by healthcare providers. For example, Chinese people tend to have a strong sense of family and tend to share their emotional issues only with their family members rather than with healthcare professionals [20]. This suggests a need for healthcare professionals to proactively assess the emotional status of Chinese patients to allow them to develop tailored care plans. Moreover, Chinese culture has been shown to play a significant role in preventing Chinese patients from discussing sexual issues with healthcare professionals [21], which highlights a need for healthcare professionals to initiate discussions with patients about sex during care delivery. The effects of culture on health needs may lead Chinese cancer patients to have different perceptions of the severity of their symptoms, including those of their sexual problems. Indeed, a previous study observed that cultural values and ethnicity can affect a patient’s experience during their illness, which in turn affects their perceptions of the experienced symptoms and their QOL [22]. Therefore, reports of sexual experiences among Western GC patients may not be applicable to their Chinese counterparts. Nurses in Chinese societies have been found to be hesitant and unprepared to provide sexuality care to GC patients because of limited resources and inadequate training, so they tend to avoid initiating discussions about sexuality with their patients [23]. Sexuality is a neglected topic even in Western countries, though most healthcare professionals regard it as an important component of nursing care [24]. Notably, despite the importance of promoting sexual health to enhance patients’ QOL, the sexual health needs and sexuality care of patients having completed GC treatment remain understudied [25]. A study on the sexual life experiences of Chinese GC patients after treatment and their perceptions of the care services provided is warranted.

We previously examined quantitatively the association and causal relationship between sexual function and QOL in Chinese patients treated for GC in a cross-sectional study with 225 subjects, and concluded that sexual function has a profound effect on their QOL and that this effect is mediated by psychosocial adjustment [26]. Nevertheless, previous reports have not included first-hand accounts of the types of sexual problems experienced by these patients or their perceptions of the quality of sexuality care provided by healthcare providers. This study aimed to qualitatively examine the effects of cancer treatment on Chinese patients treated for GC in terms of their sexual function, sexual activity, and relationships with their partner/spouse. The study also explored their perceptions regarding the information about sexuality that they received from healthcare professionals during survivorship care.

## 2. Materials and Methods

### 2.1. Design

This study had a qualitative design, and we adopted a phenomenological approach for its implementation. In this approach, researchers explore the “lived experiences” of the participants on a particular phenomenon based on their personal perceptions [27], where the phenomenon in our study is the post-treatment experiences among the participants. The study comprised a portion of our previously published quantitative study, which examined the association between sexual function and QOL among Chinese patients treated for GC in Hong Kong and complements the qualitative results of this study [26]. 

### 2.2. Participants

The participant recruitment procedures, study settings, and eligibility criteria have been reported elsewhere [26]. Briefly, the participants were recruited from a gynaecological oncology outpatient clinic at a public hospital in Hong Kong via convenience sampling in a face-to-face manner. Women who met the following criteria were considered eligible for study participation: (1) diagnosis of GC (cervical, ovarian, or uterine cancer) at least three months earlier; (2) completion of curative cancer treatment, including surgery, chemotherapy, or radiotherapy; (3) the ability to communicate in Chinese; (4) a lack of a history of any form of psychiatric disorder; and (5) non-terminal-stage disease.

Among the participants who completed data collection in our previous study [26], 21 women who were sexually active during the previous one-month period were recruited by a research nurse via purposive sampling to participate in the present qualitative study to achieve data saturation. All 21 women agreed to participate in the qualitative study.

### 2.3. Data Collection

Data were collected through individual, semi-structured interviews that lasted approximately 30 to 45 min. First, an interview guide was developed to assess the participants’ perceptions regarding the effects of cancer treatment on their sexual lives, femininity, and relationships with their partners; their views about the comprehensiveness of the sexual information provided by healthcare professionals; and their suggestions for improving sexuality care. The interviews were conducted in a quiet room at the hospital in accordance with a guide for conducting research interviews and were audio-recorded using a voice recorder. All interviews were conducted by a female research nurse who had received training from the first author (an academic researcher with a Doctor of Nursing degree and a rich clinical experience in gynaecological oncology and qualitative study). The interviewer had no prior interaction with any of the participants, and the participants had no prior knowledge of the research interests of the interviewer, namely the sexuality care of GC patients. Before the interviews, the participants were informed that the interviews would be digitally recorded. They were also advised about the anonymity of the collected data and their right to withdraw from the study without providing a reason. Informed consent was obtained from all participants before the commencing the interviews.

### 2.4. Data Analysis

A qualitative content analysis was performed to evaluate the collected qualitative data. The interview recordings were initially transcribed verbatim, and the transcripts were checked for accuracy by a research assistant. The transcripts were then read twice to identify statements pertinent to the study objectives, and the identified statements were coded and grouped into categories by the first author based on their commonality of meaning with respect to the responses to the questions set in the interview guide. Themes were then derived based on the categories generated from the coded statements. The interviews were conducted in Chinese, so the quotes and themes were translated into English by a research assistant and checked independently for accuracy by the second author.

## 3. Results

Twenty-one patients treated for GC participated in the qualitative interviews, and their demographic and clinical characteristics are presented in Table 1. Briefly, most participants were married (81.0%) and had at least one child (76.2%). They had received a diagnosis of cervical cancer (19.0%), uterine cancer (52.4%), ovarian cancer (23.8%), or a combination of uterine and ovarian cancers (4.8%). Most participants had early-stage (stage I) cancer (81.0%). For cancer treatment, most had undergone surgery alone (71.4%), whilst others had undergone chemotherapy alone (4.8%) or a combination of surgery and adjunctive therapy (23.8%).

Table 2 presents the participants’ perceptions of the effects of GC treatment on their sexual lives, femininity, and relationships with their partners and their views about the comprehensiveness of the sexual information provided by healthcare professionals. The participants’ experiences were categorised into four themes and nine subthemes.

### 3.1. Changes in Sexual Life

Overall, most participants (85.7%) reported that their sexual function had been impaired because of cancer treatment. In particular, they reported experiencing one or more gynaecological symptoms and sexual issues, including vaginal dryness, dyspareunia, failure to achieve orgasm, reduced sexual interest, and reduced sexual satisfaction. The participants also expressed a reduction in the frequency of sexual activity with their partners. The participants reported the following:

- “My sex drive has decreased, possibly because of the narrowing of my vagina. It has been very painful every time (having sex). I do not want a sexual life.” (Participant P9)
- “Sexual intercourse is quite uncomfortable. Having sex is very painful. It is too painful to endure. Therefore, I have no libido for a sexual life. My frequency of having sex is reduced.” (Participant P16)
- “I am totally unsatisfied with my sexual life because having sex makes my vagina very dry, painful, and uncomfortable. I was overwhelmed with pain. I do not want to have sex again.” (Participant P18)
- “The biggest impact is the feeling of pain when having sex. My vagina was dry owing to reduced vaginal secretions. It was really uncomfortable. I do not want to have sex again... In fact, I did not feel happy nor excited when having sex…” (Participant P20)

Some participants also expressed concerns about resuming their sex lives because of their fear of cancer recurrence:

- “The treatment has impacted my sexual life a lot. I tried having sex after completing my treatment. However, I am worried that having sex would lead to cancer recurrence, as I am concerned that sexual intercourse would lead to infections and that these infections would make the cancer return.” (Participant P19)
- “I have not dared to have sex with my partner for a long time… I always had a feeling of being traumatised, so I am concerned that having sex would affect my condition and lead to the recurrence of my ovarian cancer.” (Participant P21)

Notably, one participant had certain misconceptions about the possibility of transmitting cancer to her partner via sexual intercourse. This misconception was reported by the participant as a factor that led to the cessation of sexual activity. The participant reported the following:

- “I was informed by my neighbours that having sexual intercourse with my husband would lead to the transmission of my cancer to him. I was concerned and therefore did not dare to do have sexual intercourse.” (Participant P13)

When asked about the strategies used to address the reported sexual problems, most participants reported that they used K-Y jelly, a water-based lubricant, to treat vaginal dryness. Some reported that their doctors had recommended the use of this lubricant. Generally, the participants expressed that they felt more comfortable after applying the lubricant.

- “They recommended the application of the jelly to provide lubrication. I tried using it, and its effect was modest. I felt less pain and was more comfortable.” (Participant P20)
- “The doctor told me to use the jelly and to face the illness with a relaxed mood. I tried doing this and felt much better.” (Participant P21)

However, the participants did not report the use of any other strategies to address their sexual problems.

### 3.2. Impact on Femininity

Eleven of the 21 participants (52.4%) reported that the treatment they had undergone for GC made them feel that they had lost femininity. These participants tended to describe themselves as imperfect or incomplete women. Notably, five of these participants reported that this feeling of imperfection or incompleteness was due to the surgical removal of the uterus, which they considered to be an important symbolic feminine organ. Two of the women even reported mood problems related to the loss of their uterus. The five participants reported the following:

- “I feel sad about the loss of my uterus. I have emotional problems because of this.” (Participant P11)
- “I feel that I have lost my femininity a little bit. A complete woman should possess a uterus. I also have more frequent menopausal symptoms after the removal of my uterus. I have become more bad-tempered, and my skin is not as delicate and tender as before.” (Participant P18)
- “The loss of my uterus makes me feel like I am not a perfect woman.” (Participant P19)
- “I feel I have lost femininity because losing my uterus makes me feel like I lost the most important thing as a woman. I initially found this unacceptable…” (Participant P20)

In addition, some participants reported that, as women, they felt less attractive than they did before undergoing cancer treatment.

- “Maybe due to the effects of hormones, I feel that I am less attractive than before.” (Participant P17)

### 3.3. Relationships with the Partner/Spouse

Most participants (90.5%) reported that their relationships with their partners had not been affected negatively after the completion of cancer treatment. The participants were able to enjoy good relationships with their spouses. Interestingly, seven participants expressed that their relationships with their husbands had improved after the completion of treatment and that they now treasured each other even more. Quotes from two of these seven participants are presented below:

- “On the contrary, our relationship has become better. We take care of each other.” (Participant P14)
- “Our relationship is better than before. My husband cherishes me even more.” (Participant P16)

In contrast, two other participants reported that their relationships with their partners had been affected negatively, and they attributed this effect primarily to their inability and/or refusal to participate in sexual activity. One participant reported that pain had caused difficulty when having sexual intercourse with her husband, which then prompted her to refuse the resumption of sexual life:

- “Our relationship is fair, but it has been negatively affected. Sexual intercourse is painful, and I have become bad-tempered because of the effects of menopause. Therefore, I refuse to have sex (with my husband). My husband finds it difficult to accept this.” (Participant P18)

Another participant had even considered a divorce because she felt that she was not able to satisfy her husband:

- “The relationship has been negatively affected. I sometimes think that I cannot give him satisfaction and have considered asking for a divorce.” (Participant P19)

### 3.4. Provision of Sexual Information by Healthcare Professionals

Most participants perceived that the sex-related information provided by healthcare professionals during cancer treatment had been inadequate. Even if the professionals had provided information, it was limited to advice regarding when sexual intercourse could be resumed. The participants reported the following:

- “My doctor told me that I can resume my sexual life as normal. Apart from this, no other healthcare worker has provided me with any sex-related information.” (Participant P2)
- “The information they provided was not sufficient. The doctor just informed me that I can resume my sexual life after treatment completion. Information on other aspects was not provided.” (Participant P16)

Notably, the participants reported that healthcare professionals failed to provide any information about suggested strategies for promoting sexual health during the post-treatment period.

- “There was a lack of provision of sexual care and support during my treatment. I was only informed that I could resume my sexual life after the healing of the wound (in my vagina).” (Participant P18)
- “I feel that the sexual care provided by healthcare workers was insufficient during my cancer treatment. They did not provide me with any information on this topic. For example, they did not give me any advice about how to address sexual problems or any discomfort experienced. They did not even give me a clear account of my condition, not to mention the sexual aspects. Therefore, I am not satisfied.” (Participant P19)

Among the participants who reported receiving sexual information from healthcare professionals during the treatment phase, approximately half expressed that they only received this information after requesting it proactively. The healthcare professionals usually provided verbal advice and a leaflet containing sexual information to address the participants’ information needs. These participants reported the following:

- “(The doctor) never proactively provided the information to me. I had to ask for the information on my own initiative.” (Participant P5)
- “During my treatment, the doctors and nurses only focused on getting the surgery completed as soon as possible and did not provide any support or information on sexuality. When I attended a follow-up visit after treatment completion, I proactively asked the doctor for information about strategies to address sexual problems. The doctor provided me with a leaflet (containing sexual information), and I was informed of when I could resume sexual life and that having sex would not affect my condition.” (Participant P21)

Nevertheless, three of the participants reported that despite their desire to obtain sexual information from healthcare providers, they found it difficult to initiate a discussion on this topic because of embarrassment. This observation suggests that GC patients encounter barriers to obtaining sexual information if such information is not provided proactively by healthcare professionals. Notably, one participant even reported that she would rather search for sexual information on the Internet than request it from healthcare professionals.

- “Sexual information was not provided to me. I do not dare to ask for it, and I also do not know how to ask for it. It was embarrassing to ask.” (Participant P4)
- “There was no follow-up that addressed sexual problems during my follow-up visits with the doctor. I have not asked for it because I find it difficult to initiate the discussion on this topic. I do not know what to do. I only obtain sexual information from the Internet.” (Participant P9)
- “The doctor did not talk about it (sexual information). I feel embarrassed to ask for it.” (Participant P13)

The participants then made suggestions regarding the improvement of sexuality care, based on their perceptions regarding the limitations of the care provided. The participants recommended that healthcare professionals should initiate discussions about sexual issues proactively, at an appropriate time, and in a suitable discussion format. Four of the participants suggested that female healthcare professionals, such as nurses, would be the most suitable personnel to discuss sexual issues with GC patients. Indeed, one participant stated that she would prefer to discuss sexual issues with female healthcare professionals because it would be embarrassing to talk about sexual issues with a man.

- “It would be better for me to discuss (sexual issues) with nurses. Doctors are usually busy.” (Participant P2)
- “Female nurses or psychologists would be preferable, and it would be embarrassing to discuss this with a male doctor.” (Participant P9)

Notably, one participant expressed her preference for the discussions regarding sexual issues to be initiated by healthcare professionals, given the sensitive nature of the topic.

- “It is difficult for me to start talking about sexual issues. Healthcare workers should take the initiative to start the conversation with us.” (Participant P20)

As for the appropriate timing for such discussions, a significant proportion of the participants (42.8%) indicated that post-treatment follow-up visits would provide optimal opportunities for discussions on sexual issues with healthcare professionals. Nevertheless, two of the participants indicated that they would prefer to have these discussions at an earlier time point, before cancer treatment.

The participants suggested a variety of formats for the dissemination of sexual information to patients. The largest proportion of participants suggested that the information should be provided during individual counselling sessions (*n* = 9). Other recommended formats included health talks and seminars (*n* = 4), telephone inquiries (*n* = 1), and organised patient support groups (*n* = 1). Some participants indicated that brochures containing sexual information could be distributed to supplement these strategies.

## 4. Discussion

Overall, our data demonstrate that Chinese patients treated for GC generally suffer from impaired sexual function, a loss of femininity, and a sense of being an imperfect woman after they complete cancer treatment. Nevertheless, the participants in our study generally reported that their relationship with their partners was not severely affected by the treatment. Moreover, the participants were not satisfied with the adequacy of the sexual information they received after treatment, and they encountered barriers to initiating discussions on sexual issues with healthcare professionals.

Consistent with the results of previous studies [28,29,30], our results indicate that vaginal dryness, painful intercourse, failure to achieve orgasm, reduced sexual interest, and reduced satisfaction are commonly experienced by Chinese GC patients after treatment completion. A loss of sexual interest may be caused by a dramatic decrease in oestrogen levels due to curative cancer treatment [31], which has been shown to be associated with the occurrence of vaginal dryness and dyspareunia. These symptoms reduced the patients’ desire for sexual activities and decreased the frequency of sexual activity with their partners [32], as reported by some participants of our study. Furthermore, the patients in our study reported a poorer body image, consistent with the findings of a previous study [13], and a sense of being an imperfect woman. These perceptions can lead to other psychological issues, including anxiety and depression [10]. Indeed, a previous study showed that the sexual problems experienced by patients treated for GC are associated with depression and can reduce psychological QOL [33]. These data suggest that sexual problems and psychological symptoms form a symptom cluster in these patients, such that sexual problems are likely to occur concurrently with psychological symptoms such as depression and anxiety. Nevertheless, further studies on patients treated for GC are needed to validate this hypothesis via symptom cluster analyses.

Our findings also demonstrate that GC treatment generally has no significant influence on a patient’s relationship with their partner, regardless of the impaired sexual functioning they experience. This observation is consistent with that of an earlier study in which Chinese patients treated for GC were able to maintain good relationships with their husbands [34]. Interestingly, multiple Western studies have reported the deterioration of relationships between patients treated for GC and their partners after treatment completion [5,12,13,28,35]. This discrepancy between Western and Chinese studies in terms of the observed effects of treatment on a patient’s relationship with their partner suggests that patient ethnicity can affect perceptions on this matter. Indeed, a previous study demonstrated that Chinese people often experience a sense of strong bonding between family members [36], which is potentially attributable to the influence of Chinese culture. Given these strong family values, marital relationships would be less likely to be affected by adversities faced by a spouse. In contrast, the difficulties associated with illness would strengthen the bonds between couples. Thus, our findings further demonstrate that the patients’ ethnicity and the associated variations in cultural values should be considered in the development of care practices, as was previously suggested [37].

Consistent with the findings in previous Western studies on patients treated for GC, the Chinese GC patients in this study reported a desire for more information about sexual aspects, and most of them reported a preference for receiving this information through one-on-one counselling and discussions initiated by healthcare professionals [38,39]. Notably, these patients mostly wished to receive information about the effects of treatment on sexual function and how sexual dysfunction can be addressed [40]. However, our data indicate that such information has not been provided adequately under current practices in Hong Kong. At best, doctors only instruct their patients on the use of water-soluble lubricants to alleviate vaginal discomfort and do not provide advice regarding other sexual problems. Despite their expressed desire for more sexual information, these patients were generally reluctant to initiate discussions with healthcare professionals because of embarrassment. The above phenomena were also reported in Western studies, which indicates that healthcare professionals generally do not address patients’ sexual concerns adequately and are unlikely to initiate conversations about sexual issues [41,42]. To address these limitations of the provided care, interventions should be developed to address the sexual issues of these patients and satisfy their information needs. Ideally, these interventions would involve individual counselling and group-based seminar sessions.

Our previous study showed significant impairment of sexual function among Chinese patients treated for GC [26], and psychosocial adaptation was found to mediate the association between their sexual function and QOL. Our current study further demonstrated that these Chinese GC patients often have reduced sexual satisfaction and interest, potentially because of the experience of dyspareunia and other sexual symptoms. More importantly, we found that psychological problems among these patients could be attributable to their perception of the loss of their femininity and the sense of being an imperfect woman. Psychosocial adjustment was previously shown to mediate the relationship between sexual function and QOL, so it is likely that the implementation of psychosocial interventions to enhance the ability of patients treated for GC to cope with their perceived loss of femininity would be effective in reducing the negative effect of the loss of sexual function on their QOL. Thus, both the information and psychological needs of these patients must be addressed concurrently. Our findings therefore provide further evidence regarding the need to apply the principles of the shared care model to enhance the effectiveness of care. According to the model [18], a multidisciplinary team should be involved in the delivery of such an intervention. Whilst cancer treatment is completed by physicians, female healthcare professionals, such as nurses, are responsible for providing adequate sexual information to satisfy the patients’ information needs for sexual health promotion, in addition to providing psychological care to address their emotional issues. Larger-scale studies to investigate whether the delivery of interventions using such a shared care approach would enhance the QOL of patients treated for GC could be valuable. Such studies would provide an evidence base for the implementation of psychosocial interventions tailored to address the loss of femininity as an integral part of survivorship care for these patients.

Another point of interest for discussion is that not all of the participants reported impaired sexual function or a loss of femininity after they had received GC treatment. For example, about 86% of the subjects expressed that they experienced impaired sexual function, and only 52% of them reported a loss of femininity. These data imply that GC treatment likely has different levels of effect on the participants’ sexual function and sexual satisfaction. Such difference could potentially be attributed to a number of factors that could confound the perception of the participants. For example, our sample consisted of participants who had either completed treatment for less than 12 months or over 12 months (Table 1). As longer duration after GC treatment completion could potentially result in participants being more adapted to the detrimental effect of the treatment, those having completed treatment for a longer duration could have perceived a better sexual health than those who had just completed treatment. A mixture of participants with various time since treatment completion in our sample could therefore result in variations in perceived sexual experience among them, leading to heterogeneity in the collected qualitative data. Moreover, the wide variation of age in our sample could also contribute to the variations in the participants’ reports on their sexual experience. Indeed, while some participants were aged below 45, a significant proportion of them (38%) were postmenopausal women aged over 55 (Table 1). These older postmenopausal participants would have stopped producing and releasing oestrogen from their ovaries, while their younger counterparts were still likely to be actively undergoing female hormonal production. With oestrogen known to positively affect sexual function, arousal, and satisfaction in women [43,44,45], younger participants were more likely to have reported better sexual function and higher levels of sexual satisfaction compared to the older participants. Following this line of argument, the type of cancer treatment that the participants had could also play a part in the variations of study findings. In our study, most of the participants had undergone surgery for the removal of the uterus with ovaries and fallopian tubes, although one underwent chemotherapy only (Table 1). With ovaries being the primary site of oestrogen production, these participants were likely to have suffered more sexual problems compared to those who had undergone other forms of treatment such as chemotherapy, due to their inability to produce oestrogen to counter the detrimental effect of the GC treatment on their sexual function and desire. Patients having undergone surgery would therefore be more likely to perceive a reduced sexual function, desire, and/or satisfaction. Moreover, as the removal of reproductive organs was shown to contribute to a loss of perceived femininity among women [46], undergoing surgery by patients in our study could have had a more profound impact on their femininity than other treatments. However, since only one participant had not undergone surgery in our study (Table 1), the effect of treatment modalities received on the participants’ sexual experience should be minimal. Although largely speculative, the above factors could have contributed to the observed differences in the effect of GC treatment on the participants’ outcomes. Future studies should consider the utilisation of samples comprising women at a particular menopausal status and those having a similar time since treatment completion. 

### 4.1. Implications for Healthcare Practice

**Figure 1:**
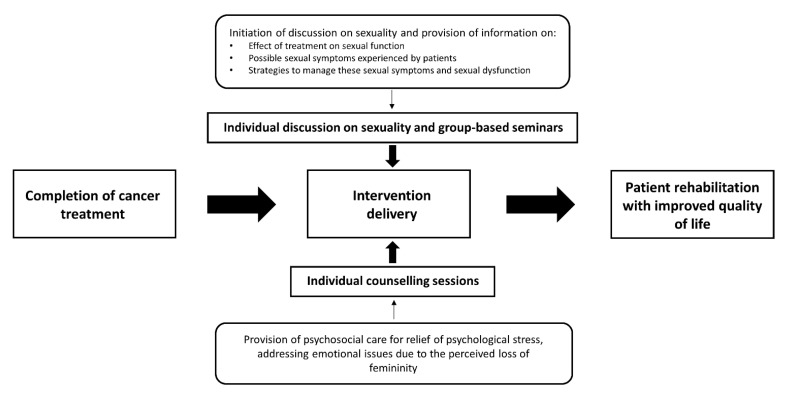
Proposed model of care for Chinese gynaecological cancer survivors.

The reported experiences of sexual problems and psychological symptoms in our study clearly demonstrate that patients treated for GC would benefit from a better understanding about these issues and treatment strategies. Interventions delivered via a shared care approach, as described in Figure 1, should be implemented to address both the information and psychological needs of these patients. These interventions would increase the patients’ knowledge about sexual issues, improve their self-efficacy in self-management of their symptoms, and promote their psychological health. These interventions should be led by female healthcare professionals, such as nurses, to increase the comfort of the patients during discussions about their sexual issues.

### 4.2. Limitations

This study has several limitations. First, our sample purely consisted of GC patients who were ethnically Chinese. With the potential difference in culture between the Chinese and other world populations, and the potential effect of culture on perceived QOL [47], our findings, which are related to sexual quality of life, are not likely to be generalisable to GC patients of other ethnic origins. Second, our study was of a cross-sectional design, where the comparison of outcomes at pre-treatment and post-treatment and the assessment of longitudinal changes of these outcomes were not possible. There was also no “control group” that comprised participants at their pre-treatment stage, thus we cannot confirm whether the sexual health outcomes reported by the participants were really attributed to the GC treatment received. Third, our sample comprised women of various ages, who had completed treatment for various periods of time. As indicated above, these factors could be confounding factors on women’s sexual experience. Fourth, our findings on whether GC treatment has impacted patients’ relationships with their partners were primarily based on the reports by the patients of this study in their responses to questions regarding their current relationship with their partners and whether such relationship had been affected by the treatment. In this study, we did not collect interview data from the patients’ partners, leading to the inability to verify the reports provided by these patients on these issues. Owing to the above limitations, the findings of this study should be interpreted with caution.

## 5. Conclusions

Chinese patients treated for GC often experience a range of sexual symptoms and express a considerable need for information about strategies to address these symptoms. Healthcare practices should therefore aim to satisfy this need by providing the required information. Nurse-led interventions should be implemented to enhance the patients’ awareness of sexual symptom management and alleviate their psychological symptoms. These interventions should seek to reduce the psychosocial suffering of the patients and improve their QOL.

## Figures and Tables

**Table 1:** Participant characteristics (*n* = 21).

Characteristics	*n* (%)
**Age (years)**	
≤45	6 (28.6%)
46–55	7 (33.3%)
>55	8 (38.1%)
**Education**	
Below high school	10 (47.6%)
High school or above	11 (52.3%)
**Monthly household income (HK$)**	
<20,000	11(52.3%)
≥20,000	10 (47.6%)
**Marital status**	
Married	17 (81.0%)
Single with regular sexual partner	3 (14.3%)
Single without regular sexual partner/divorced/widowed	1 (4.8%)
**Had children**	
No	5 (23.8%)
Yes	16 (76.2%)
**Country of Origin**	
Hong Kong	12 (57.1%)
Mainland China	9 (42.9%)
**Years of residence in Hong Kong**	
<3 years	8 (38.1%)
3–6 years	8 (38.1%)
≥6 years	5 (23.8%)
**Type of gynaecological cancer diagnosed**	
Cervical	4 (19.0%)
Uterine	11 (52.4%)
Ovarian Uterine + ovarian	5 (23.8%) 1 (4.8%)
**Stage of cancer**	
Stage I	17 (81.0%)
Stage II	4 (19.0%)
**Treatment modalities**	
Surgery only	15 (71.4%)
Chemotherapy only	1 (4.8%)
Surgery + adjunctive therapy	5 (23.8%)
**Time since the completion of treatment**	
≤12 months	5(23.8%)
≥12 months	16(76.2%)
Hysterectomy	
**Total hysterectomy**	17 (81.0%)
Partial hysterectomy No	3 (14.3%) 1 (4.8%)
**Menopause**	
No	7 (33.3%)
Yes	12 (57.1%)
Decline to disclose	2 (9.5%)

Data are presented as frequency (%).

**Table 2:** Themes and subthemes generated in this study.

Themes	Subthemes
Changes in sexual life	Impaired sexual function Misconceptions about resuming sexual life
Impact on femininity	Loss of femininity Psychological distress regarding the loss of the uterus
Relationship with partner/spouse	Closer relationship with the partner after treatment Risk of discord in the relationship due to sexual dysfunction
Provision of sexual information by healthcare professionals	Limited provision of information about sexual issues Desire to receive more information about sexual issues Recommendation for proactive discussions initiated by healthcare professionals

## Data Availability

The data presented in this study are available on request from the corresponding author (C.W.H.C.).
